# Supplementary figures and images for: Choosing the best route: Comparative optimization of wheat transformation methods for improving yield by targeting TaARE1-D with CRISPR/Cas9
Source: PLoS One. 2026 Feb 9;21(2):e0342491. doi: 10.1371/journal.pone.0342491 (PMC12885284; doi:10.1371/journal.pone.0342491)

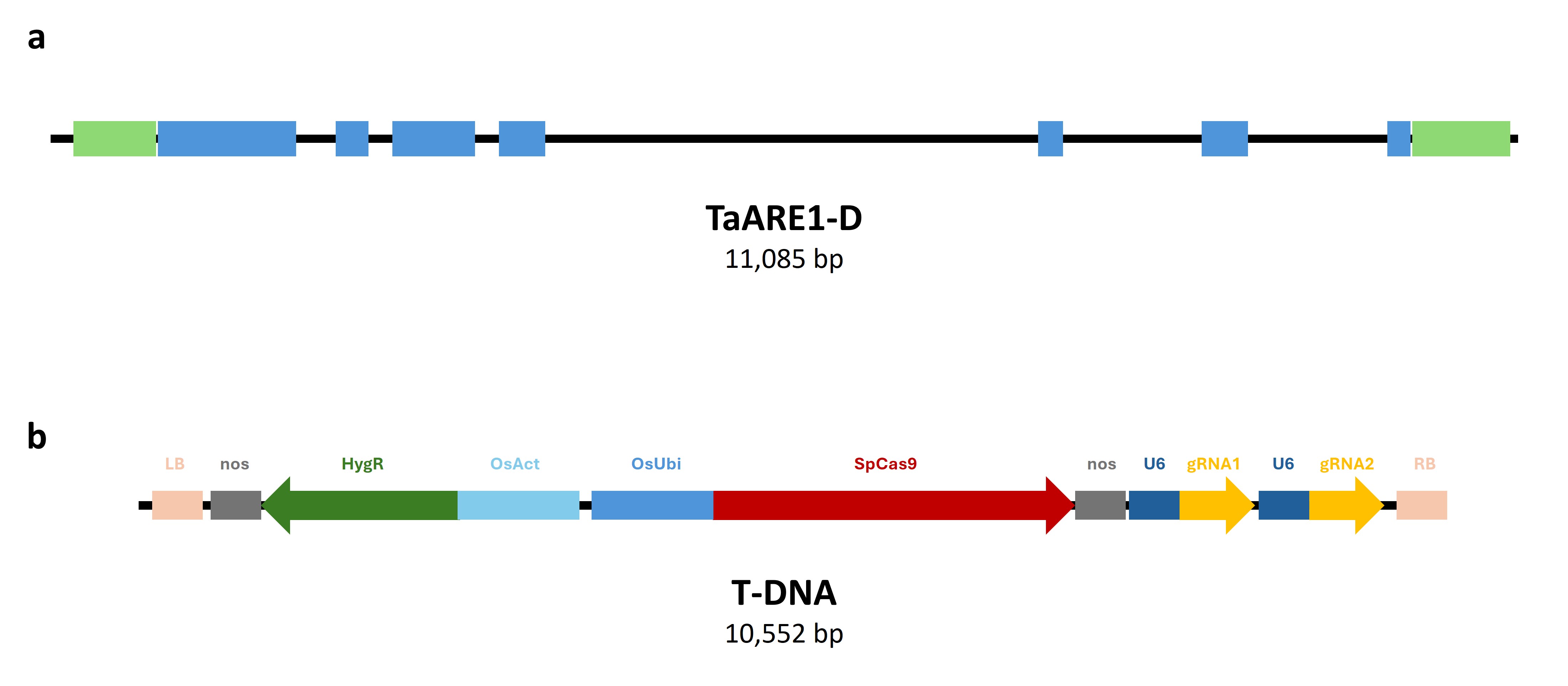

Supplement: S1 Fig — (JPG) [file pone.0342491.s001.jpg]

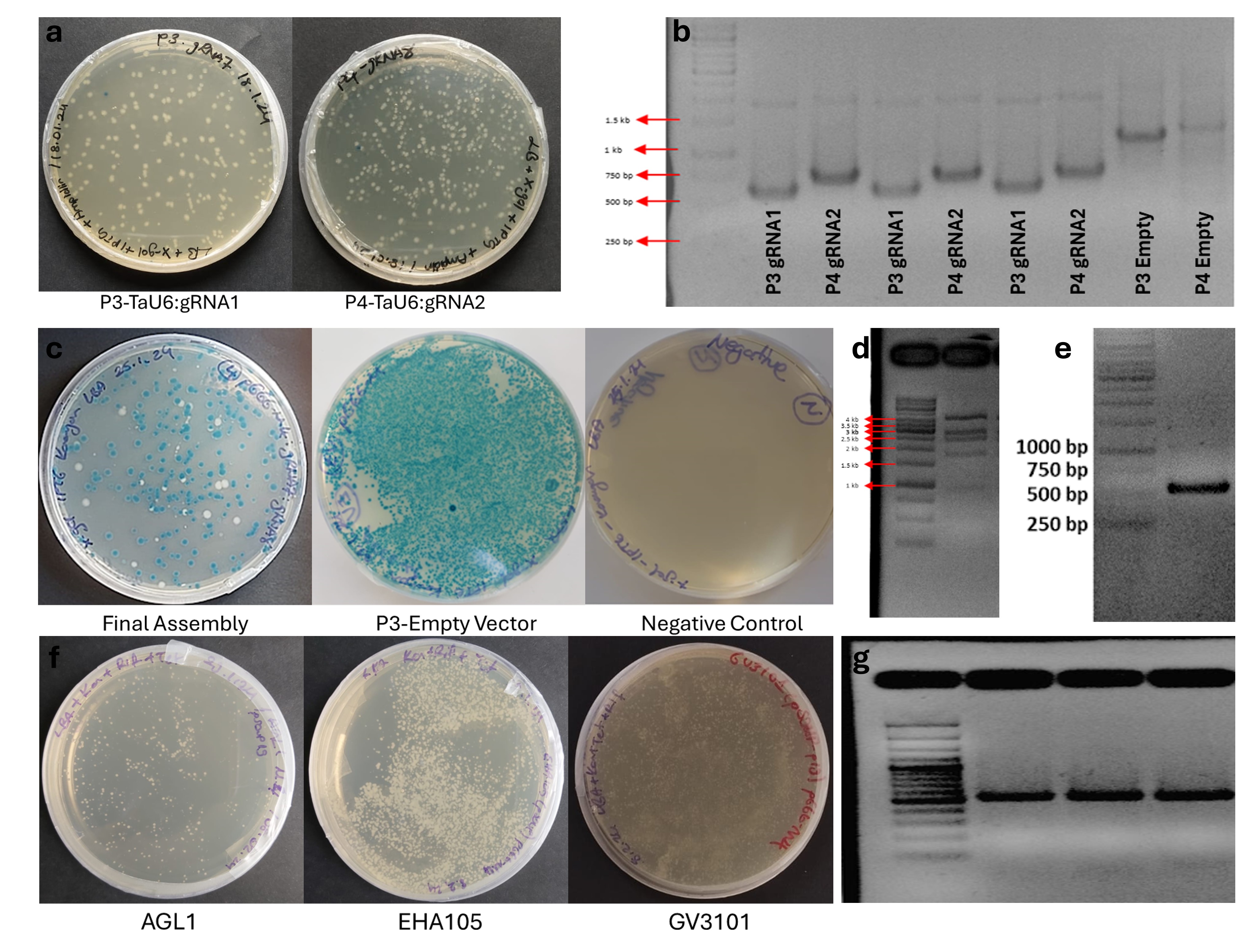

Supplement: S2 Fig — (JPG) [file pone.0342491.s002.jpg]

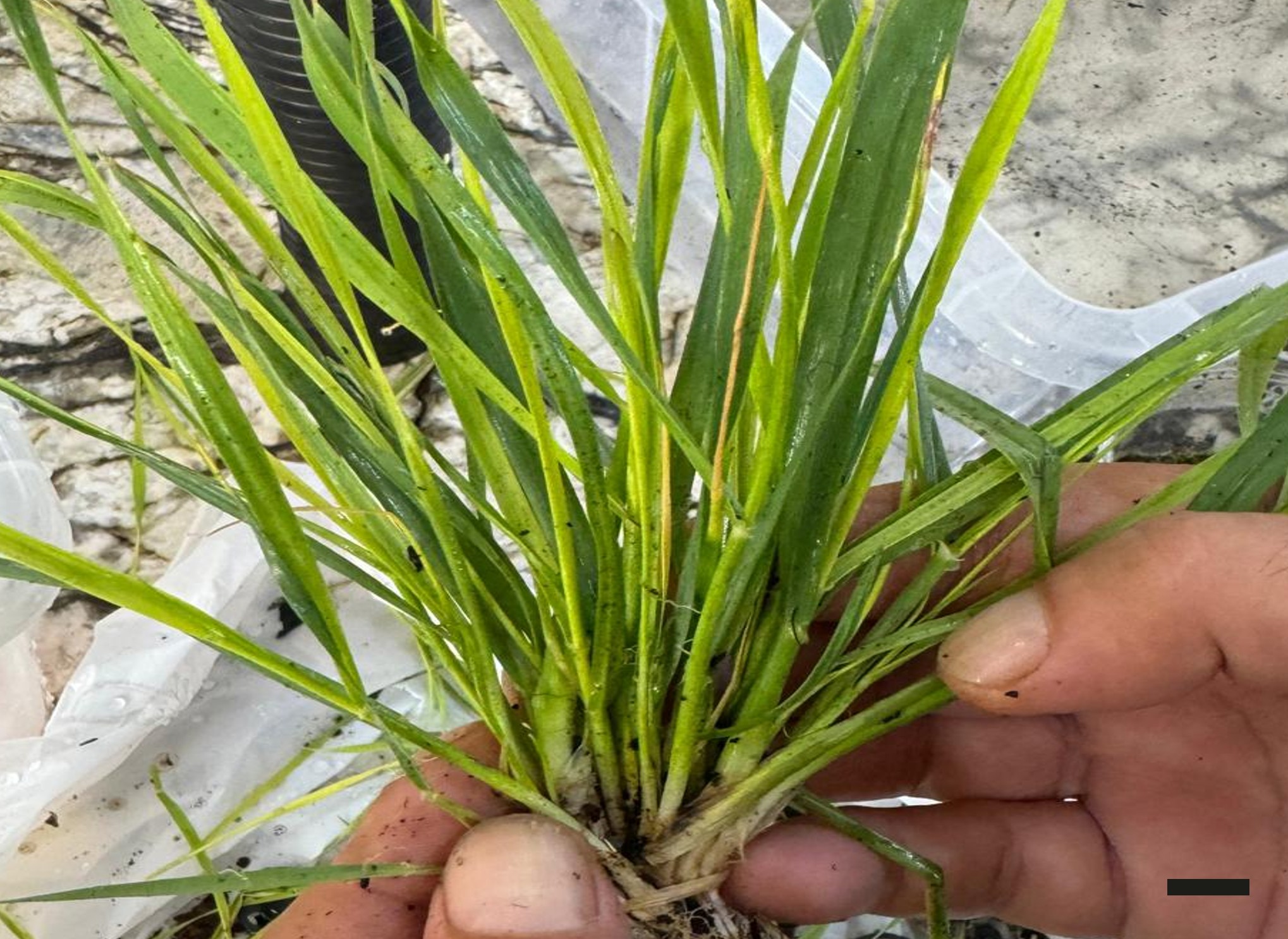

Supplement: S3 Fig — (JPG) [file pone.0342491.s003.jpg]

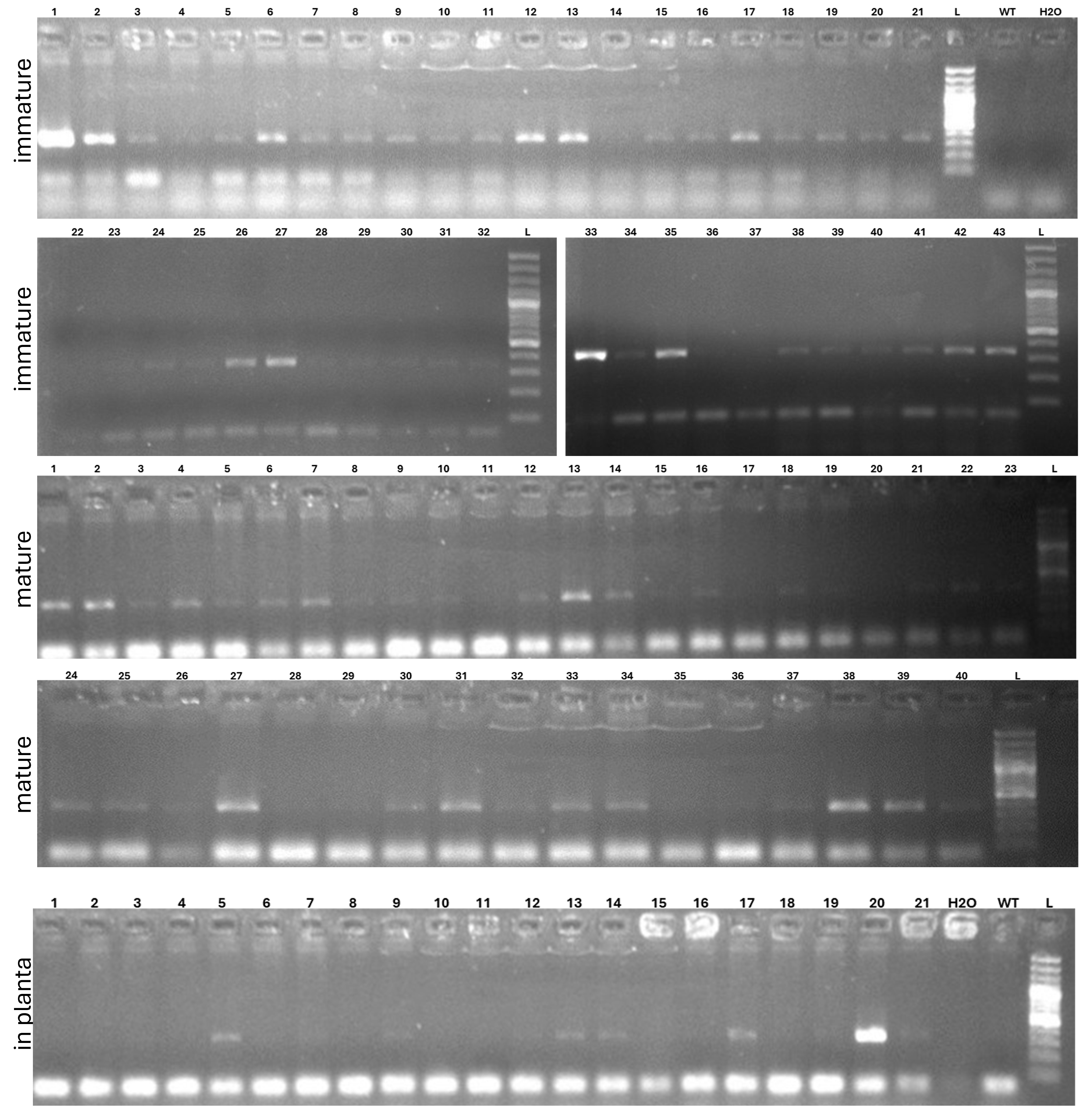

Supplement: S4 Fig — (TIF) [file pone.0342491.s004.tif]

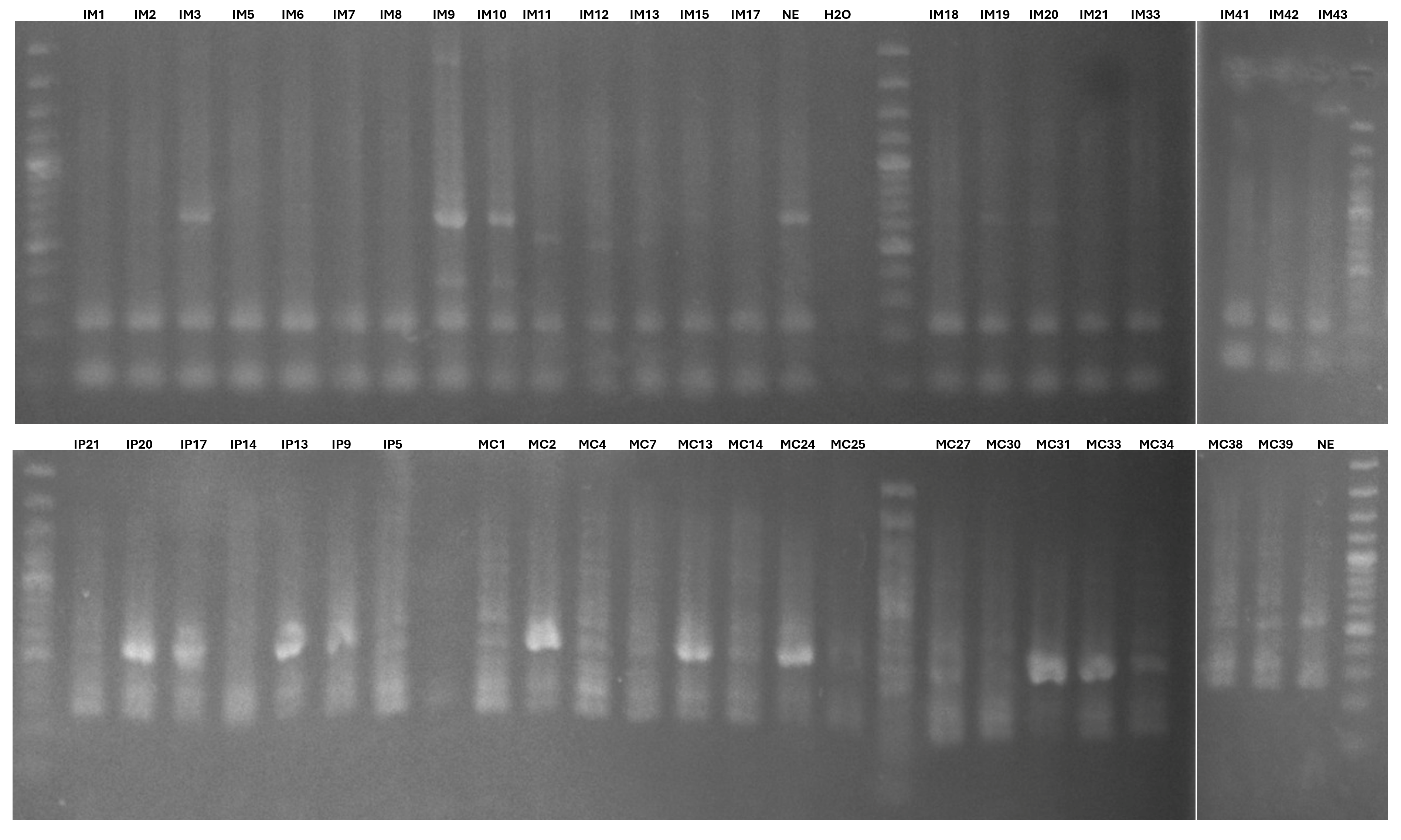

Supplement: S5 Fig — Absence of amplification indicates CRISPR/Cas9-induced mutations at the gRNA1 target site. (TIF) [file pone.0342491.s005.tif]

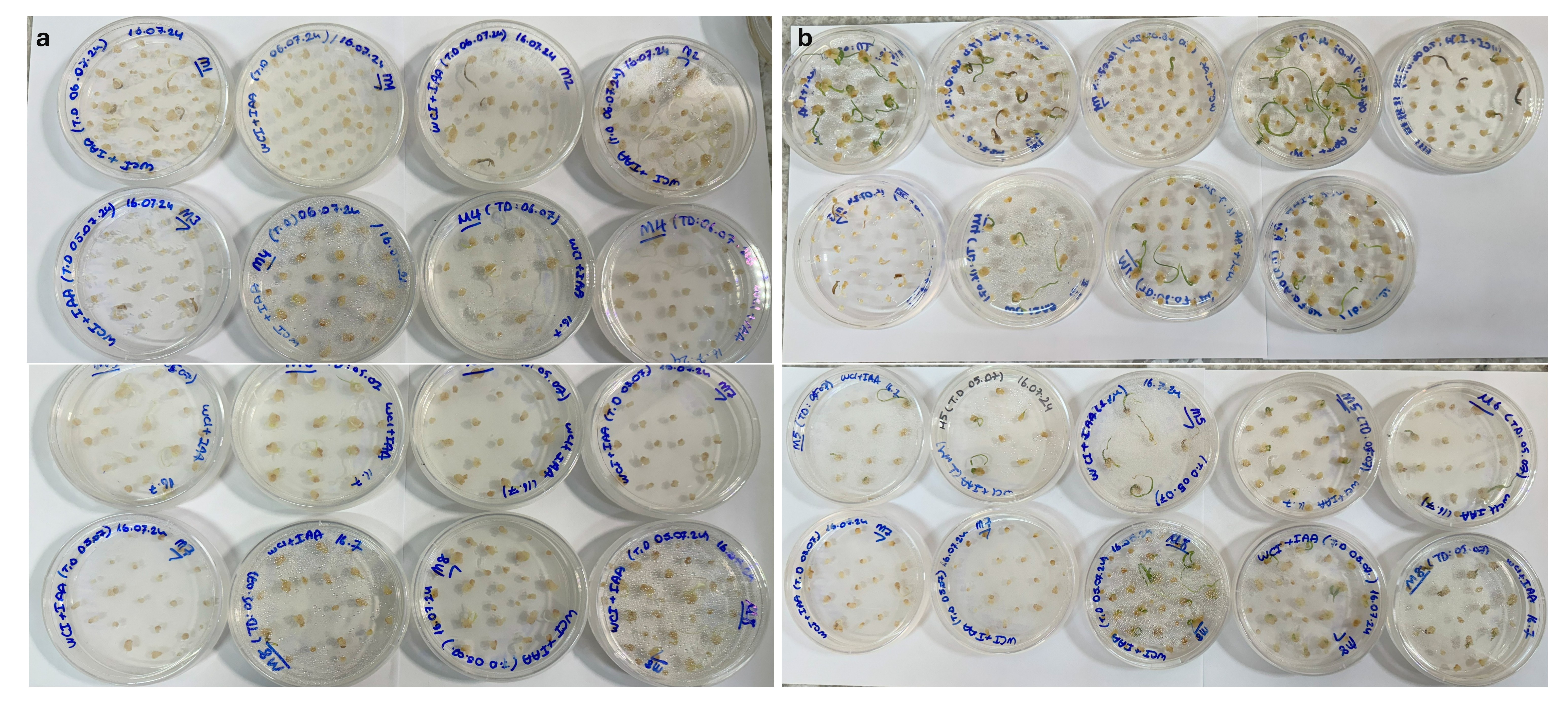

Supplement: S6 Fig — (JPG) [file pone.0342491.s006.jpg]

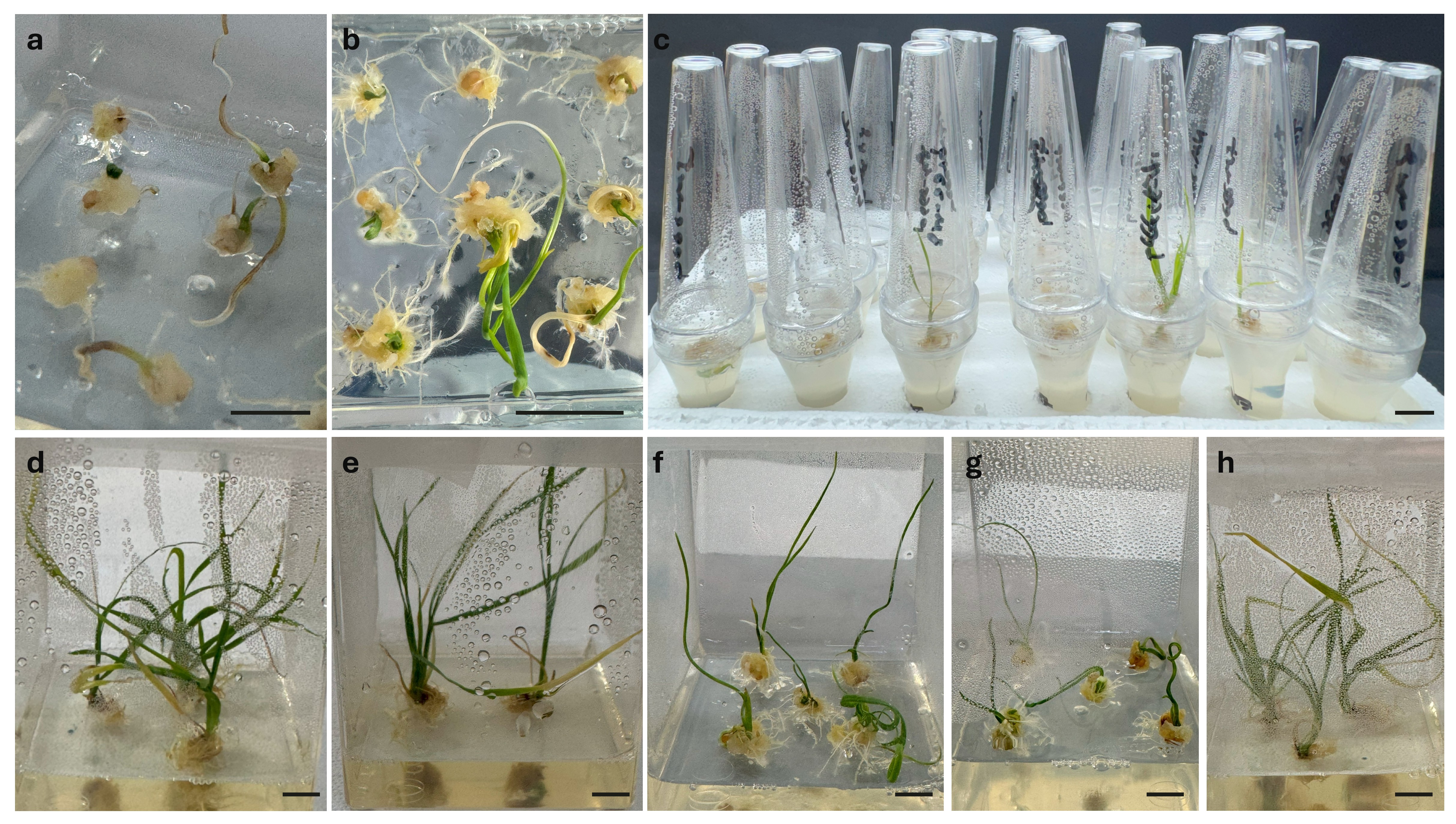

Supplement: S7 Fig — (JPG) [file pone.0342491.s007.jpg]

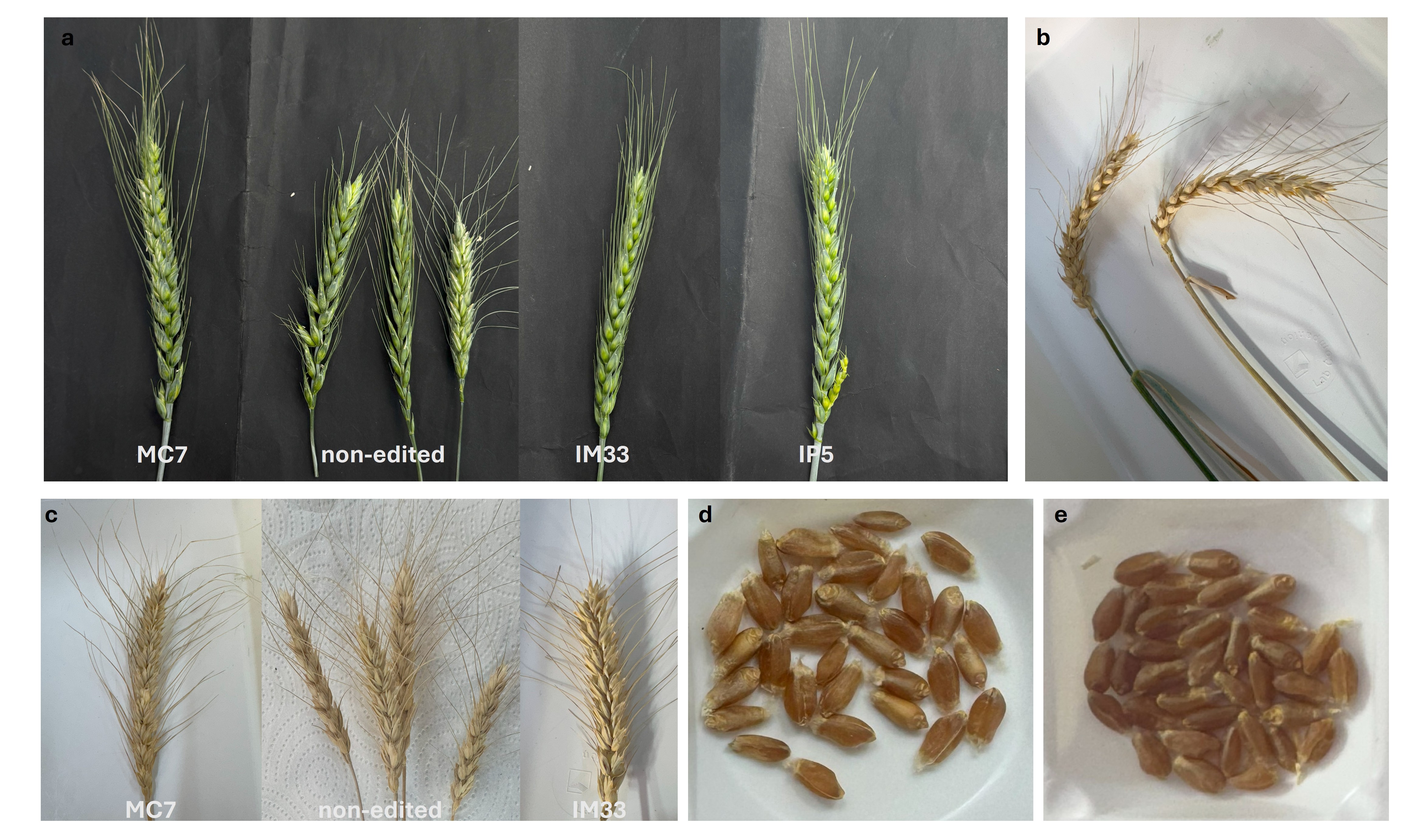

Supplement: S8 Fig — (JPG) [file pone.0342491.s008.jpg]

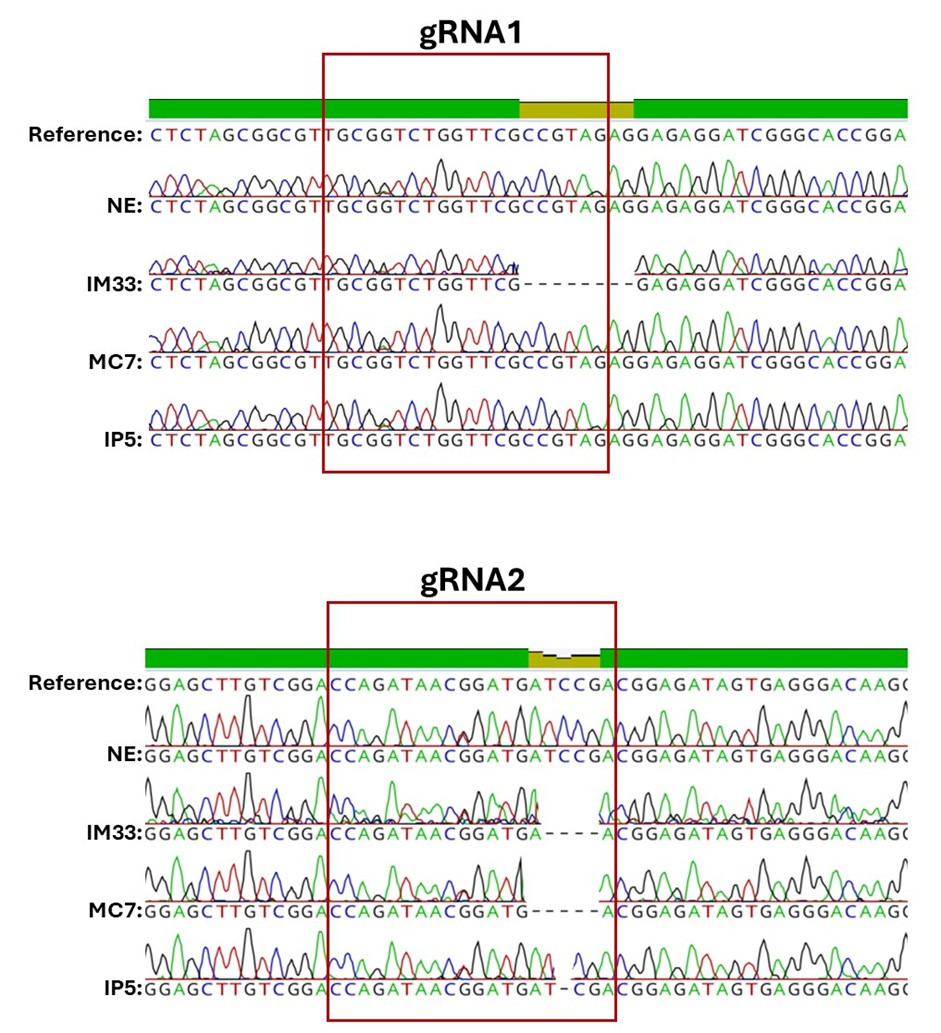

Supplement: S9 Fig — (JPG) [file pone.0342491.s009.jpg]
