# Supplementary material for: Choosing the best route: Comparative optimization of wheat transformation methods for improving yield by targeting TaARE1-D with CRISPR/Cas9
Source: PLoS One. 2026 Feb 9;21(2):e0342491. doi: 10.1371/journal.pone.0342491 (PMC12885284; doi:10.1371/journal.pone.0342491)
Supplement: S1 Appendix — (DOCX) [file pone.0342491.s019.docx]

>TaARE1DReference

AGCGGCGTTGCGGTCTGGTTCGCCGTAGAGGAGAGGATCGGGCACCGGAGGGTTTGCGCATGCAAGATGTTCGATGTCGGTCCCCAGAGGAGGAGGGTGGGGAGGCGCCTGGTGGGTTTTGCCAAGAAGAGGAGGCGTTCCAAGAGGCAGCAGCCATGGTGGAAGGCGTGGTTCTCTGATTGGAACGATGAGGAAGAGAGCCTCGCCGGCTGGAGGGAGGATGATGAATTGCTCCAGCAGGTTGTTAGCAACGAAGACCTGTCGGAGGATGACAAGTTTCAGACGTGGAAGAGCAAGGCCGAGGCGATTGTCGACCTGCGGGAAGCCCAGCAGGATGCCGAAAATGCAGAAGGGCGGTCATGGGAGGATTGGATAGGTTGGGGCAGCACGTCCGGCGATGGTGATTGGGGCGGGGGTGGGAGCTTGTCGGACCAGATAACGGATGATCCGACGGAGATAGTGAGGGAC

>NE

CTCTAGCGGCGTTGCGGTCTGGTTCGCCGTAGAGGAGAGGATCGGGCACCGGAGGGTTTGCGCATGCAAGATGTTCGATGTCGGTCCCCAGAGGAGRAGGGTGGGGAGGCGCCTGGTGGGTTTTGCCAAGAAGAGGAGGCGTTCCAAGAGGCAGCAGCCATGGTGGAAGGCGTGGTTCTCTGATTGGAACGATGAGGAAGAGAGCCTCGCCGGCTGGAGGGAGGATGATGAATTGCTCCAGCAGGTTGTTAGCAACGAAGACCTGTCGGAGGATGACAAGTTTCAGACGTGGAAGAGCAAGGCCGAGGCGATTGTCGACCTGCGGGAAGCCCAGCAGGRTGCCGAAAATGCAGAAGGGCGGTCATGGGAGGATTGGATAGGTTGGGGCAGCACGTCCGGCGATGGTGATTGGGGCGGGGGTGGGAGCTTGTCGGACCAGATAACGGATGATCCGACGGAGATAGTGAGGGACAAGGGCATCGCTGAAGCTTTTAGGGACTCTATTGATGAAGATTACAACGACATGTTGTTTGAGGA

>IM33T1

CTAGCGGCGTTGCGGTCTGGTTCGGAGAGGATCGGGCACCGGAGGKTTCGCACATGCAAGATGTTCGATGTCGGTCCCCAGAGGAGRAGGGTGGGGAGGCGCCTGGTGGGTTTTGCCAAGAAGAGGAGGCGTTCCAAGAGGCAGCAGCCATGGTGGAAGGSGTGGTTCTCTGATTGGAACGGTGAGGAAGAGAGCCTCGCCGGCTGGAGGGAGGATGATGAATTGCTCCAGCAGGTTGTTAGCAACGAAGACCTGTCGGAGGATGACAAGTTTCAGACGTGGAAGAGCAAGGCCGAGGCGATTGTCGACCTGCGGGAAGCCCAGCAGGRTGCCGAAAATGCARAAGGGCGGTCATGGGAGGATTGGATAGGTTGGGGCAGCACGTCCGGCGATGGTGATTGGGGCGGGGGTGGGAGCTTGTCGGACCAGATAACGGATGAACGGAGATA

>IM21

TAGCGGCGTTGCGGTCTGGTTCGCTAGAGGAGAGGATCGGGCACCGGASGKTTTGCGCATGCAAGATGTTCGATGTCGGTCCCCAGAGGAGRAGGGTGGGGAGGCGCCTGGTGGGTTTTGCCAAGAAGAGGAGGCGTTCCAAGAGGCAGCAGCCATGGTGGAAGGCGTGGTTCTCTGATTGGAACGATGAGGAAGAGAGCCTCGCCGGCTGGAGGGAGGATGATGAATTGCTCCAGCAGGTTGTTAGCAACGAAGACCTGTCGGAGGATGACAAGTTTCAGACGTGGAAGAGCAAGGCMGAGGCGATTGTCGACCTGCGGGAAGCCCAGCAGGRTGCCGAAAATGCAGAAGGKSGGTCATGGGAGGATTGGATAGGTTGGGGCAGCACRTCCGGCGATGGTGAYTGGGGCGGGGGTGGGAGCTTGTCGGACCAGATAACGGATGATGACGGAGATAGTGAGGGACAAGGGCATCGCTGAAGCTT

>IM8

GTCAGCTCTAGCGGCGTTGCGGTCTGGTTCGCCCGTAGAGGAGAGGATCGGGCACCGGAGGKTTTGCGMATGCAAGATGTTCGATGTCGGTCCCCAGAGGAGRAGGGTGGGGAGGCGCCTGGTGGGTTTTGCCAAGAAGAGGAGGCGTTCCAAGAGGCAGCAGCCATGGTGGAAGGCGTGGTTCTCTGATTGGAACGATGAGGAAGAGAGCCTCGCCGGCTGGAGGGAGGATGATGAATTGCTCCAGCAGGTTGTTAGCAACGAAGACCTGTCGGAGGATGACAAGTTTCAGACGTGGAAGAGCAAGGCCGAGGCGATTGTCGACCTGCGGGAAGCCCAGCAGGATGCCGAAAATGCAGAAGGGCGGTCATGGGAGGATTGGATAGGTTGGGGCAGCACGTCCGGCGATGGTGATTGGGGCGGGGGTGGGAGCTTGTCGGACCAGATAACGGATGATCCGACGGAGATAGTGAGGGACAAGGGCATCGCTGAAGCTTTTAGGGACTCTAWTGATGAAGATTACAACGACATGTTGTTTGAGGACCGGGTTTTTCTATACGCTTCGACGAAATCGGTACTTCTAGCACTA

>IM7

TGGGTCAGCTCTAGCGGCGTTGCGGTCTGGTTCGCCGTAGAGGAGAGGATCGGGCACCGGAGGKYTTGCGCATGCAAGATGTTCGATGTCGGTCCCCAGAGGAGRAGGGTGGGGAGGCGCCTGGTGGGTTTTGCCAAGAAGAGGAGGCGTTCCAAGAGGCAGCAGCCATGGTGGAAGGCGTGGTTCTCTGATTGGAACGATGAGGAAGAGAGCCTCGCCGGCTGGAGGGAGGATGATGAATTGCTCCAGCAGGTTGTTAGCAACGAAGACCTGTCGGAGGATGACAAGTTTCAGACRTGGAAGAGCAAGGCCGAGGCGATTGTCGACCTGCGGGAAGCCCAGCAGGRTGCCGAAAATGCAGAAGGGCGGTCATGGGAGGATTGGATAGGTTGGGGCAGCACGTCCGGCGATGGTGAYTGGGGCGGGGGTGGGAGCTTGTCGGACCAGATAACGGATGATCGACGGAGATAGTGAGGGACAAGGGCATCGCTGAAGCTTT

>IM6

CTAGCGGCGTTGCGGTCTGGTTCGCCGAGAGGAGAGGATCGGGCACCGGAGGKTTTGCGCATGCAAGATGTTCGATGTCGGTCCCCAGAGGAGRAGGGTGGGGAGGCGCCTGGTGGGTTTTGCCAAGAAGAGGAGGCGTTCCAAGAGGCAGCAGCCATGGTGGAAGGCGTGGTTCTCTGATTGGAACGATGAGGAAGAGAGCCTCGCCGGCTGGAGGGAGGATGATGAATTGCTCCAGCAGGTTGTTAGCAACGAAGACCTGTCGGAGGATGACAAGTTTCAGACGTGGAAGAGCAAGGCMGAGGCGATTGTCGACCTGCGGGAAGCCCAGCAGGRTGCCGAAAATGCAGAAGGGCGGTCATGGGAGGATTGGATAGGTTGGGGCAGCACRTCCGGCGATGGTGAYTGGGGCGGGGGTGGGAGCTTGTCGGACCAGATAACGGATGATCCGACGGAGATAGTG

>MC7

GGGTCAGCCTCTAGCGGCGTTGCGGTCTGGTTCGCCGTAGAGGAGAGGATCGGGCACCGGAGGKTTTGCGCATGCAAGATGTTCGATGTCGGTCCCCAGAGRGAGRAGGGTGGGGAGGCGCCTGGTGGGTTTTGCCAAGAAGAGGAGGCGTTCCAAGAGGCAGCAGCCATGGTGGAAGGCGTGGTTCTCTGATTGGAACGATGAGGAAGAGAGCCTCGCCGGCTGGAGGGAGGATGATGAATTGCTCCAGCAGGTTGTTAGCAACGAAGACCTGTCGGAGGATGACAAGTTTCAGACGTGGAAGAGCAAGGCCGAGGCGATTGTCGACCTGCGGGAAGCCCAGCAGGRTGCCGAAAATGCAGAAGGGCGGTCATGGGAGGATTGGATAGGTTGGGGCAGCACGTCCGGCGATGGTGATTGGGGCGGGGGTGGGAGCTTGTCGGACCAGATAACGGATGACGGAGATAGTGAGGGACAAGGGCATCGCTGAAGCTTT

>MC8

GGGTCAGCTCTAGCGGCGTTGCGGTCTGGTTCGCCGTAGAGGAGAGGATCGGGCACCGGAGGKYTTKYGCATGCAAGATGTTCGATGTCGGTCCCCAGAGGAGRAGGGTGGGGAGGCGCCTGGTGGGTTTTGCCAAGAAGAGGAGGCGTTCCAAGAGGCAGCAGCCATGGTGGAAGGCGTGGTTCTCTGATTGGAACGATGAGGAAGAGAGCCTCGCCGGCTGGAGGGAGGATGATGAATTGCTCCAGCAGGTTGTTAGCAACGAAGACCTGTCGGAGGATGACAAGTTTCAGACGTGGAAGAGCAAGGCCGAGGCGATTGTCGACCTGCGGGAAGCCCAGCAGGRTGCCGAAAATGCAGAAGGGCGGTCATGGGAGGATTGGATAGGTTGGGGCAGCACGTCCGGCGATGGTGATTGGGGCGGGGGTGGGAGCTTGTCGGACCAGATAACGGATGATCGACGGAGATAGTGAGGGACAAGGGCATCGCTGAAGCTTTTAGGGACTCTAWTGATGAAGATTA

>MC14

GGTCAGCTCTAGCGGCGTTGCGGTCTGGTTCGCCTAGAGGAGAGGATCGGGCACCGGAGGKTTTGCGCATGCAAGATGTTCGATGTCGGTCCCCAGAGGAGRAGGGTGGGGAGGCGCCTGGTGGGTTTTGCCAAGAAGAGGAGGCGTTCCAAGAGGCAGCAGCCATGGTGGAAGGCGTGGTTCTCTGATTGGAACGATGAGGAAGAGAGCCTCGCCGGCTGGAGGGAGGATGATGAATTGCTCCAGCAGGTTGTTAGCAACGAAGACCTGTCGGAGGATGACAAGTTTCAGACGTGGAAGAGCAAGGCMGAGGCGATTGTCGACCTGCGGGAAGCCCAGCAGGRTGCCGAAAATGCAGAAGGGCGGTCATGGGAGGATTGGATAGGTTGGGGCAGCACRTCCGGCGATGGTGAYTGGGGCGGGGGTGGGAGCTTGTCGGACCAGATAACGGATGATCCGACGGAGATAGTGAGGGACAAGGG

>MC25

CTAGCGGCGTTGCGGTCTGGTTCGCCGAGAGGAGAGGATCGGGCACCGGAGGKTTTGYGCATGCAAGATGTTCGATGTCGGTCCCCAGAGGAGRAGGGTGGGGAGGCGCCTGGTGGGTTTTGCCAAGAAGAGGAGGCGTTCCAAGAGGCAGCAGCCATGGTGGAAGGCGTGGTTCTCTGATTGGAACGATGAGGAAGAGAGCCTCGCCGGCTGGAGGGAGGATGATGAATTGCTCCAGCAGGTTGTTAGCAACGAAGACCTGTCGGAGGATGACAAGTTTCAGACGTGGAAGAGCAAGGCCGAGGCGATTGTCGACCTGCGGGAAGCCCAGCAGGRTGCCGAAAATGCAGAAGGGCGGTCATGGGAGGATTGGATAGGTTGGGGCAGCACGTCCGGCGATGGTGATTGGGGCGGGGGTGGGAGCTTGTCGGACCAGATAACGGATGATCCGACGGAGATAGTGAGGGACAAGGGCATCGCTGA

>MC30

AGCGGCGTTGCGGTCTGGTTCGCCGAGAGGAGAGGATCGGGCACCGGAGGKTTTGCGCATGCAAGATGTTCGATGTCGGTCCCCAGAGGAGRAGGGTGGGGAGGCGCCTGGTGGGTTTTGCCAAGAAGAGGAGGCGTTCCAAGAGGCAGCAGCCATGGTGGAAGGCGTGGTTCTCTGATTGGAACGATGAGGAAGAGAGCCTCGCCGGCTGGAGGGAGGATGATGAATTGCTCCAGCAGGTTGTTAGCAACGAAGACCTGTCGGAGGATGACAAGTTTCAGACGTGGAAGAGCAAGGCCGAGGCGATTGTCGACCTGCGGGAAGCCCAGCAGGRTGCCGAAAATGCAGAAGGGCGGTCATGGGAGGATTGGATAGGTTGGGGCAGCACGTCCGGCGATGGTGATTGGGGCGGGGGTGGGAGCTTGTCGGACCAGATAACGGATGATCCGACGGAGATAGTGAGGGACAAGGG

>IP5

GCGGCGTTGCGGTCTGGTTCGCCGTAGAGGAGAGGATCGGGCACCGSAGGGARTGCGMATGCAAGATGTTCGATGTCGGTCCCCAGAGGAGGAGGGTGGGGAGGCGCCTGGTGGGTTTTGCCAAGAAGAGGAGGCGTTCCAAGAGGCAGCAGCCATGGTGGAAGGCGTGGTTCTCTGATTGGAACGATGAGGAAGAGAGCCTCGCCGGCTGGAGGGAGGATGATGAATTGCTCCAGCAGGTTGTTAGCAACGAAGRCCTGTCGGAGGATGACAAGTTTCARACGTGGAAGAGCAAGGCMGAGGCGATTGTCGACCTGCGGGAAGCCCAGCAGGRTGCCGAAAATGCAGAAGGGCGGTCATGGGAGGATTGGATAGGTTGGGGCAGCACGTCCGGCGATGGTGATTGGGGCGGGGGTGGGAGCTTGTCGGACCAGATAACTGATGATCGACGGAGATAGTGAGGGACAAGGGCAM

>IP14

GCGGCGTTGCGGTCTGGTTCGCCAGAGGAGAGGATCGGGCACCGGAGGGTTTGCGCATGCAAGATGTTCGATGTCGGTCCCCAGAGGAGGAGGGTGGGGAGGCGCCTGGTGGGTTTTGCCAAGAAGAGGAGGCGTTCCAAGAGGCAGCAGCCATGGTGGAAGGCGTGGTTCTCTGATTGGAACGATGAGGAAGAGAGCCTCGCCGGCTGGAGGGAGGATGATGAATTGCTCCAGCAGGTTGTTAGCAACGAAGACCTGTCGGAGGATGACAAGTTTCAGACGTGGAAGAGCAAGGCCGAGGCGATTGTCGACCTGCGGGAAGCCCAGCAGGATGCCGAAAATGCAGAAGGGCGGTCATGGGAGGATTGGATAGGTTGGGGCAGCACGTCCGGCGATGGTGATTGGGGCGGGGGTGGGAGCTTGTCGGACCAGATAACGGATGATCCGACGGAGATA

>IP21

CGGCGTTGCGGTCTGGTTCGCCGTAGAGGAGAGGATCGGGCACCGGAGGKTTTGCGCATGCAAGATGTTCGATGTCGGTCCCCAGAGGAGGAGGGTGGGGAGGCGCCTGTGGGTTTTGCCAAGAAGAGGAGGCGTTCCAAGAGGCAGCAGCCATGGTGGAAGGCGTGGTTCTCTGATTGGAACGATGAGGAAGAGAGCCTCGCCGGCTGGAGGGAGGATGATGAATTGCTCCAGCAGGTTGTTAGCAACGAAGACCTGTCGGAGGATGACAAGTTTCAGACGTGGAAGAGCAAGGCCGAGGCGATTGTCGACCTGCGGGAAGCCCAGCAGGATGCCGAAAATGCAGAAGGGCGGTCATGGGAGGATTGGATAGGTTGGGGCAGCACGTCCGGCGATGGTGATTGGGGCGGGGGTGGGAGCTTGTCGGACCAGATAACGGATGATCCGACGGAGATAGTGAGGGACAAG

>IM33T0

CTCTAGCGGCGTTGCGGTCTGGTTCGGAGAGGATCGGGCACCGGAGGGTTTGCGCATGCAAGATGTTCGATGTCGGTCCCCAGAGGAGRAGGGTGGGGAGGCGCCTGGTGGGTTTTGCCAAGAAGAGGAGGCGTTCCAAGAGGCAGCAGCCATGGTGGAAGGSGTGGTTCTCTGATTGGAACGGTGAGGAAGAGAGCCTCGCCGGCTGGAGGGAGGATGATGAATTGCTCCAGCAGGTTGTTAGCAACGAAGACCTGTCGGAGGATGACAAGTTTCAGACGTGGAAGAGCAAGGCCGAGGCGATTGTCGACCTGCGGGAAGCCCAGCAGGRTGCCGAAAATGCARAAGGGCGGTCATGGGAGGATTGGATAGGTTGGGGCAGCACGTCCGGCGATGGTGATTGGGGCGGGGGTGGGAGCTTGTCGGACCAGATAACGGATGAACGGAGATAGTGAGGGACAAGGGCATCSCTGAARCTTTTTAGGGACTCTATTGATGAAGATTACAACGACATG

>MC7T0

CTCTAGCGGCGTTGCGGTCTGGTTCGCCGTAGAGGAGAGGATCGGGCACCGGAGGGTTTGCGCATGCAAGATGTTCGATGTCGGTCCCCAGAGRGAGRAGGGTGGGGAGGCGCCTGGTGGGTTTTGCCAAGAAGAGGAGGCGTTCCAAGAGGCAGCAGCCATGGTGGAAGGCGTGGTTCTCTGATTGGAACGATGAGGAAGAGAGCCTCGCCGGCTGGAGGGAGGATGATGAATTGCTCCAGCAGGTTGTTAGCAACGAAGACCTGTCGGAGGATGACAAGTTTCAGACGTGGAAGAGCAAGGCCGAGGCGATTGTCGACCTGCGGGAAGCCCAGCAGGRTGCCGAAAATGCAGAAGGGCGGTCATGGGAGGATTGGATAGGTTGGGGCAGCACGTCCGGCGATGGTGATTGGGGCGGGGGTGGGAGCTTGTCGGACCAGATAACGGATGACGGAGATAGTGAGGGACAAGGGCATCGCTGAAGCTTTTAGGGACTCTATTGATGAAGATTACAACGACATGTTGTTTGAGGACCGGGTTTTTCTA

>IP5T0

CTAGCGGCGTTGCGGTCTGGTTCGCCGTAGAGGAGAGGATCGGGCACCGGAGGGTTTGCGCATGCAAGATGTTCGATGTCGGTCCCCAGAGGAGRAGGGTGGGGAGGCGCCTGGTGGGTTTTGCCAAGAAGAGGAGGCGTTCCAAGAGGCAGCAGCCATGGTGGAAGGCGTGGTTCTCTGATTGGAACGATGAGGAAGAGAGCCTCGCCGGCTGGAGGGAGGATGATGAATTGCTCCAGCAGGTTGTTAGCAACGAAGACCTGTCGGAGGATGACAAGTTTCAGACGTGGAAGAGCAAGGCCGAGGCGATTGTCGACCTGCGGGAAGCCCAGCAGGRTGCCGAAAATGCAGAAGGGCGGTCATGGGAGGATTGGATAGGTTGGGGCAGCACGTCCGGCGATGGTGATTGGGGCGGGGGTGGGAGCTTGTCGGACCAGATAACGGATGATCGACGGAGATAGTGAGGGACAAGGGCATCGCTGAAGCTTTTAGGGACTCTAWTGATGAAGATTACAACGACATGTTGTTTGAGGACCGGGTTTTTCTATA
